# The Potential of *Bacillus* Species as Probiotics in the Food Industry: A Review

**DOI:** 10.3390/foods13152444

**Published:** 2024-08-02

**Authors:** Jessie Payne, Danielle Bellmer, Ravi Jadeja, Peter Muriana

**Affiliations:** 1 Department of Animal and Food Science, Oklahoma State University, Stillwater, OK 74078, USA; ravi.jadeja@okstate.edu (R.J.); peter.muriana@okstate.edu (P.M.); 2 Robert M. Kerr Food and Agricultural Products Center, Oklahoma State University, Stillwater, OK 74078, USA; danielle.bellmer@okstate.edu; 3 Department of Biosystems and Agricultural Engineering, Oklahoma State University, Stillwater, OK 74078, USA

**Keywords:** *Bacillus*, probiotics, stability, food processing, *Lactobacillus*, *Bacillus subtilis*, *Bacillus coagulans*

## Abstract

The demand for probiotics is increasing, providing opportunities for food and beverage products to incorporate and market these foods as a source of additional benefits. The most commonly used probiotics belong to the genera of *Lactobacillus* and *Bifidobacterium*, and traditionally these bacteria have been incorporated into dairy products, where they have a wider history and can readily survive. More recently, there has been a desire to incorporate probiotics into various food products, including baked goods. In recent years, interest in the use of *Bacillus* species as probiotics has greatly increased. The spores of various *Bacillus* species such as *Bacillus coagulans* and *Bacillus subtilis*, have significantly improved viability and stability under harsher conditions during heat processing. These characteristics make them very valuable as probiotics. In this review, factors that could affect the stability of *Bacillus* probiotics in food products are highlighted. Additionally, this review features the existing research and food products that use *Bacillus* probiotics, as well as future research opportunities.

## 1. Introduction

Probiotics have gained global interest due to their recognized benefits in improving gut microflora, with emerging data suggesting they offer additional health advantages. Probiotics are used in various industries, including pet supplements, sports nutrition, dietary supplements, and food and beverages. This increased awareness of their health benefits has positively impacted the probiotic sector, which is projected to grow by 14% between 2023 and 2030, with the market size reaching USD 77 billion in 2022 [1].

Probiotics are defined as “live microorganisms, which when administered in adequate amounts confer a health benefit on the host” [2]. To be considered a probiotic, an organism must meet four key criteria [3]: it must be a microorganism, alive at the time of consumption, administered in sufficient quantities (at least 10^6^ to 10^9^ cfu/g), and demonstrate beneficial effects on the health of a host [4].

The most common probiotic microorganisms are found in the genera *Lactobacillus* and *Bifidobacterium*. These species have a long history of safe use and are recognized as Generally Regarded AS Safe (GRAS) by the United States Food and Drug Administration (FDA). However, probiotics must endure harsh conditions during production, storage, and throughout their passage through the gastrointestinal (GI) tract [5]. *Lactobacillus* and *Bifidobacterium* can be particularly sensitive to the stomach’s low pH and bile salts [6]. Additionally, factors such as food components, food additives, oxygen content, redox potentials, moisture levels, water activity, storage temperatures, pH, titratable acidity, and packaging conditions can influence their viability [7]. Given the susceptibility of *Lactobacillus* and *Bifidobacterium* to various conditions, alternatives to these probiotics are necessary.

*Bacillus* has been suggested as an alternative to *Lactobacillus* and *Bifidobacterium* probiotics. *Bacillus* is closely related to *Lactobacillus* [8]. Due to their ability to form stable spores, Bacilli can withstand food processing and storage conditions better than *Lactobacillus* or *Bifidobacterium*, making *Bacillus* a suitable probiotic [8]. The spore-forming ability of *Bacillus* enhances its resistance to thermal treatment, allowing it to survive cooking processes; it can also endure harsh processing conditions such as heat, pH changes, salt, and desiccation, and withstand storage conditions that are often detrimental to vegetative cells. Several *Bacillus* species, including *Bacillus subtilis*, *Bacillus cereus*, *Bacillus clausii*, *Bacillus coagulans*, and *Bacillus licheniformis*, have been extensively studied [8]. Using *Bacillus* as a probiotic offers numerous potential benefits for developing a wider range of food products as probiotic carriers. Spore-forming bacteria can tolerate higher temperatures and lower pH environments during food processing, and they are better able to survive the harsh conditions within the GI tract [9]. Although spore-forming bacteria like *Bacillus* have become increasingly popular, only a few researchers have evaluated their viability through different food processes and storage conditions [10,11,12,13,14,15,16,17,18,19].

Numerous *Bacillus* species are now available as commercial probiotic supplements and are being incorporated into a wide array of food products. However, there is limited knowledge about the stability and longevity of *Bacillus* probiotic cultures in different food matrices. Factors influencing the viability of probiotic organisms include product attributes such as water activity, processing time and temperature, and the compatibility of probiotics with the food matrix composition. This review article addresses the stability of *Bacillus* probiotics concerning food matrices (such as carrier matrices, carbohydrates, fat content, and water activity), food processes (including drying, baking, and temperature), storage conditions, and sensory acceptability.

The review specifically focuses on current research concerning *Bacillus* probiotics and their stability during various food processes, food matrices, and storage conditions. An extensive review of probiotic research was conducted through peer-reviewed journals, bulletins, and critical reviews. Additionally, probiotic manufacturing companies such as Kerry Probiotics, Deerland Probiotics, Sanzyme Biologics, and LactoSpore were contacted for further insights.

## 2. General Probiotics

Microbiota balance is the oldest proposed benefit of probiotics. However, probiotics now offer advantages for human health beyond their nutritional effects [20]. They work by promoting the growth of “good” bacteria in the gut, which enhances the immune system’s efficiency in combating potential pathogenic bacteria [21]. For example, some probiotics have been shown to modulate the immune response in individuals with acute and chronic conditions, such as GI distress or irritable bowel disease (IBS) [20].

Most probiotic microorganisms share similar health benefits and general characteristics. These include improvements in digestive health, a reduction of pathogenic bacteria, an increased turnover of enterocytes, and the production of short-chain fatty acids [5]. In contrast, effects related to immunology, neurology, and endocrinology tend to be more strain-specific [5]. Recent research has indicated that probiotics might mitigate the negative effects of antibiotics [22]. Despite their many benefits, incorporating probiotics in food products presents challenges for ensuring humans receive these health benefits effectively.

Probiotics must be “administered” or consumed, either via encapsulation or added to food products. There are two primary methods for incorporating probiotics into food: they can either be grown into the final product through fermentation or added directly into the food product before processing. During fermentation, the growth of lactic acid bacteria produces lactic acid, which serves as the main antimicrobial agent and is often used for food fermentations. Although lactic acid bacteria are tolerant to these antimicrobials for a short period, they do not survive long-term in acidic environments, which limits their shelf-life viability during extended storage periods in fermented products. On the other hand, food manufacturers can add probiotics directly to the food product.

Incorporating probiotics by directly adding them to food is challenging as these probiotics must face harsh conditions during production and after consumption. They need to survive not only in the GI tract but also through various production processes and storage conditions [5]. Numerous attempts to include different strains of *Lactobacillus* and *Bifidobacterium* in baked products such as bread and cakes have resulted in significant losses of probiotic viability during baking [19,20,21,22,23,24,25,26]. To address this issue, the food industry has explored solutions such as microencapsulation and edible films.

Microencapsulation involves coating probiotic cells with a substance that protects them until they are released into the intestine [24,27,28,29,30,31]. This technique helps preserve sensitive strains from the food matrix until consumption. Edible films also aim to protect probiotics against storage conditions and the harsh environment of the GI tract [28]. However, both microencapsulation and edible films are relatively complicated to prepare and come with additional costs. An alternative approach is to use less sensitive, spore-forming bacteria, such as those from the *Bacillus* genus, which have shown greater resilience to harsh conditions compared to sensitive vegetative cells.

## 3. *Bacillus* as Probiotics

*Bacillus* is a Gram-positive, rod-shaped, aerobic, or facultative aerobic, spore-forming bacterium [32]. It belongs to the genus Bacilli bacteria within the family Bacillaceae. The popularity of *Bacillus* as a probiotic has increased, partly due to its stability during the processing and storage of food and pharmaceutical preparations, making it a suitable ingredient for health-promoting formulations [8]. Additionally, *Bacillus* is naturally associated with the fermentation of various foods, including soy, locust beans, maize, rice, and natto, aligning with current trends favoring “natural” products. *Bacillus*’s association with “natural” products has increased its use in both human and animal contexts.

Recently, the use of the *Bacillus* species as probiotics for humans and as an alternative to antibiotics for animals has significantly risen. This increase is partially due to the widely demonstrated benefits of *Bacillus*, which include its role as a therapeutic agent for Gastrointestinal (GI) disorders, urinary tract infections, antimicrobial production, enzyme production, and immune modulation [8]. More than 795 antibiotics have been identified from *Bacillus* bacteria [33]. The rising issue of antibiotic resistance has driven interest in alternative bacterial infection therapies, such as pro- and prebiotics [34]. However, *Bacillus* strains that produce antibiotics should not be used to prevent the development of GI antibiotic-resistant bacteria, and thus, proposed *Bacillus* probiotics should be screened for antibiotic production.

Several health benefits are attributed to the *Bacillus* genus. Some of these benefits focus on GI health, while others support overall immune system well-being [35]. *Bacillus* enhances “good” microflora, contributing to benefits such as the competitive exclusion of pathogens, increased nutritional value, the alleviation of dietary intolerance and allergies, anti-inflammatory and immune-modulating effects, the improvement of gut function, and a reduced risk of cardiovascular disease and cancer (see Table 1) [27,36]. Additionally, documented effects include the regulation of intestinal microbial homeostasis and the stimulation of local and systemic immune responses [37]. These are just some of the many benefits of using Bacillus probiotics instead of *Lactobacillus* of *Bifidobacterium* bacteria.

*Bacillus*’s potential health benefits along with its tolerance to hostile environments enhance its suitability as a functional food ingredient. In contrast, *Lactobacillus* and *Bifidobacterium* bacteria are known for their low heat resistance [26]. Most currently used probiotics are sensitive to environmental conditions; as a result, processing and storage often reduce the viability of these strains [38,39,40]. Utilizing spores has been shown to improve the viability and stability of the probiotics, particularly under the harsher conditions encountered during heat processing. Additionally, spores help reduce the economic burden on manufacturers by minimizing the loss of viability of bacteria during production processes [38].

**Figure 1:**
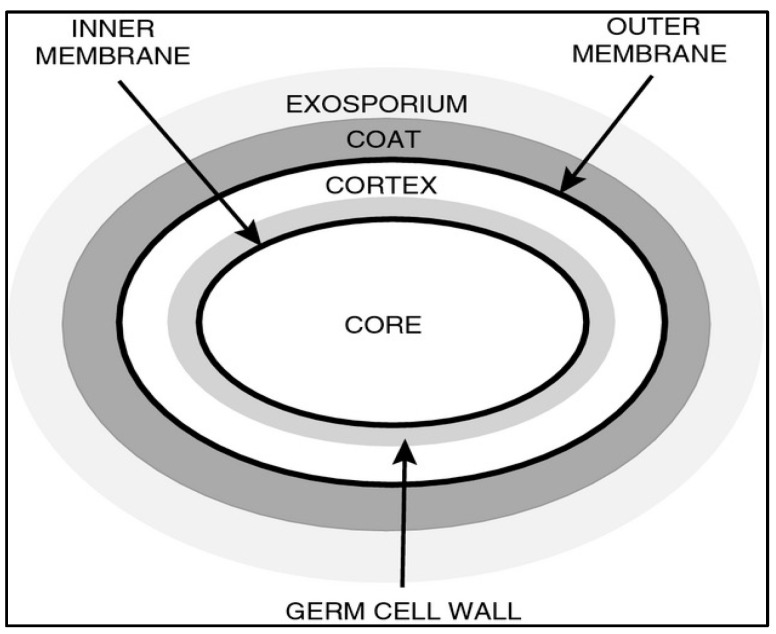
The structure of a *Bacillus* spore. Note. The different sections of a standard *Bacillus* spore are shown and labeled. Some spores do not have the Exosporium but will have additional spore coat layers in which the outermost layer will be deemed the “crust”. Modified from [44].

A spore is a dormant, non-reproductive structure produced by spore-forming microorganisms (Figure 1). The robust thermal resistance of spore-forming bacteria during food processing and digestion makes them highly valuable as probiotics. Research indicates that these bacterial spores exhibit significantly greater resistance to thermal effects, drying, freezing, toxic chemicals, and radiation compared to their vegetative cells [41]. Moreover, spores in an aqueous environment can withstand temperatures approximately 40 °C higher than their vegetative counterparts of the same strain [42]. *Bacillus* spores remain dormant for years but can rapidly germinate when provided with suitable conditions [43].

The structure of spores plays a crucial role in enhancing probiotic viability under harsh conditions. Key structural components include the outer membrane, a thick proteinaceous coat that detoxifies reactive chemicals [44]. Additionally, the cortex, composed of a rigid cell wall material, provides structural integrity and resistance to physical damage. An impermeable inner membrane further protects the spore’s coat from external factors [44]. Figure 1 illustrates the structure of a *Bacillus* spore, highlighting its various layers.

Spores of *Bacillus* bacteria undergo rapid transformation from the inactive form to the vegetative form after exposure to nutrients and moisture, such as in the intestinal tract [41]. These characteristics enhance their value as probiotics. Studies have demonstrated that *Bacillus* spores germinate in the GI tract of rabbits, dogs, and mice [32,45,46,47,48] under conditions that closely mimic human physiological environments [46], including germination in both the jejunum and ileum [45].

Another significant advantage of *Bacillus* spores as probiotics is their ability to withstand high-temperature conditions encountered during food processing. Although heat treatments typically trigger spore germination, it remains unclear whether spores or vegetative cells persist after these processes. Recent research evaluated the viability of eight *Bacillus* strains across various food processing operations—pasteurization, baking, and drying at temperatures ranging from 65 °C to 180 °C [10]. During baking at 180 °C for 20 min, most strains exhibited a reduction in counts of approximately 2 logs CFU/g. Despite exposure to high heat, *Bacillus* spores can maintain viability in bread and bakery products [33], a feat not achievable by most vegetative probiotics. Additionally, probiotic spores offer an extended shelf life compared to products containing vegetative cells [32], making them highly suitable for use in processed functional foods.

Today, a wide range of *Bacillus* strains are available as commercial probiotic supplements and are integrated into various food products (Table 2). Among the extensively studied species—such as *Bacillus subtilis*, *Bacillus cereus*, *Bacillus clausii*, *Bacillus coagulans*, and *Bacillus licheniformis*—many commercial probiotics consist of *Bacillus cereus*, *Bacillus clausii*, *Bacillus coagulans*, *Bacillus licheniformis*, *Bacillus subtilis*, or combinations thereof (Table 2). Each species exhibits unique capabilities to thrive under diverse manufacturing and storage conditions [38], with *Bacillus coagulans* and *Bacillus subtilis* recognized for their probiotic significance.

Among hundreds of known *Bacillus* species, only *Bacillus coagulans* and *Bacillus subtilis* are widely recognized as probiotics suitable for human consumption [41]. Studies conducted by [6,49] have shown that *Bacillus subtilis* ATCC 9799 and *Bacillus coagulans* GBI-30 6086, cause no adverse effects or illnesses [50]. Selected strains of *Bacillus coagulans* and *Bacillus subtilis* are increasingly incorporated into various food products due to their ability to survive gut conditions, resist bile and acid, and produce beneficial compounds for human health [51]. These strains’ capacity to form spores further enhances their suitability as probiotics.

### 3.1. Bacillus subtilis

*Bacillus subtilis* belongs to the genus *Bacillus* within the family Bacillaceae. It is a Gram-positive, rod-shaped bacterium capable of forming spores and can thrive aerobically or facultatively anaerobically. The spores of *B. subtilis* exhibit remarkable resistance to temperature fluctuations, pH variations, gastric acid, and bile encountered in the GI tract [22].

Certain strains of *B. subtilis* are known for their beneficial effects, including the competitive exclusion of pathogens from the gut. These strains can produce a diverse array of antimicrobial peptides, such as bacteriocins, and are non-toxigenic [34]. Oral administration of *B. subtilis* has been shown to stimulate the immune response, leading to the increased production of antibodies (IgG and IgA) and interaction with macrophages [22]. These immune responses contribute to anti-inflammatory effects, alleviate diarrhea, and promote normalization of intestinal flora [22].

*B. subtilis* is typically considered a member of the gut microbiota and a less common commensal organism. It frequently enters the human gastrointestinal tract via food, water, and air [22]. Studies have identified *B. subtilis* as comprising a diverse population within the GI tract, equipped with traits that enhance survival in this environment. Research also suggests a bi-modal lifecycle for *Bacillus* strains, indicating their ability to sporulate and germinate within the GI tract. Germinated *B. subtilis* DE111 cells have been observed in the small intestines of humans [52], and studies in mice demonstrate that *B. subtilis* spores can germinate and re-sporulate in the gut [22].

*Bacillus subtilis* DE111, a probiotic strain developed by Deerland Enzymes and Probiotics, has achieved GRAS (generally recognized as safe) status from the FDA. This designation confirms its safety for human consumption. Beyond safety, DE111 has demonstrated probiotic activities distinct from other *B. subtilis* strains, including the production of metabolites that aid nutrient absorption [53]. DE111 spores exhibit high heat resistance (over 100 °C), tolerate a wide pH range, and germinate efficiently in the gut [53]. This strain maintains a higher proportion of spore cells compared to vegetative cells, enhancing its resilience to stressors during manufacturing or GI transit. Furthermore, DE111 has been shown to support a healthy gut microbiome [42]. However, additional research is needed to fully understand its long-term viability and stability.

### 3.2. Bacillus coagulans

*Bacillus coagulans*, first isolated from spoiled canned milk in 1915 by Hammer [43], was initially classified as a strain of *Lactobacillus* but reclassified in 1974 due to its spore-forming abilities [6]. It is a Gram-positive, spore-forming, rod-shaped bacteria known for producing lactic acid [11]. *B. coagulans* thrives optimally at temperatures between 35 and 50 °C and pH levels of 5.5 and 6.5.

Many *B. coagulans* produced today are GRAS, as extensive studies have demonstrated their safety and lack of mutagenic, clastogenic, or genotoxic effects [6]. *B. coagulans* strains vary widely in enzymatic activities [54], with some producing lactic acid, lipase, alpha-amylase, alpha-galactosidase, or beta-galactosidase, depending on the strain [54]. Notably, strains like BC30™ (*Bacillus coagulans* GBI-30, 6086) produce lactic acid and alpha-amylase and have been extensively studied for their health benefits and incorporated into numerous products [7].

Health benefits attributed to *Bacillus* strains range from improved GI health to fostering the growth of beneficial gut microbiota. The spores of *B. coagulans* strain BC30™ possess a resilient integument primarily composed of proteins, which enables them to withstand gastric acid and bile salts for effective delivery to the intestines [37]. Studies have demonstrated that *B. coagulans* strain BC30™, due to its spore-forming ability, effectively alleviates symptoms associated with GI disorders such as irritable bowel syndrome (IBS), rheumatoid arthritis, and intestinal gas [12]. Moreover, *B. coagulans* regulates the host’s microbiota, promoting the restoration of beneficial bacteria and competitively excluding pathogens [41]. It also exhibits antimicrobial activity through the production of a bacteriocin-like substance named coagulin [55], thereby maintaining intestinal microbiota balance.

*B. coagulans*, specifically *B. coagulans* GBI-30 6086, have been recognized for their ability to survive in the GI tract and improve GI disorders. It aids in the absorption and utilization of proteins and sugars from the diet by producing various enzymes [33,37,41], which is particularly beneficial for individuals with sugar intolerance, including lactose intolerance. *B. coagulans* help alleviate lactose intolerance symptoms by digesting lactose early in the GI tract.

Recent research has expanded the incorporation of *B. coagulans* into a variety of functional foods available in the market, including pasta, chocolate, and ice cream [43]. Originally used primarily in liquid products, its application has broadened due to its resilience during various manufacturing processes. Studies have shown that BC30™ (*B. coagulans* GBI-30 6086) maintains viability in baked goods even after storage, qualifying it as a viable probiotic in functional foods [13]. It has also demonstrated resilience through milk pasteurization treatments without losing its probiotic properties [56].

Despite recent taxonomic changes proposing *B. coagulans* to be included in the genus *Weizmannia* (*Weizmannia coagulans*) [57], and subsequently in the genus *Heyndrickxia* (*Heyndrickxia coagulans*) [58], its status as a valuable probiotic remains unchanged. *B. coagulans*, particularly strains like GBI-30 6086, continue to grow in popularity due to their proven health benefits for the GI tract and their ability to withstand various manufacturing processes, thereby expanding their potential applications in functional foods.

## 4. Stability of Probiotics

Developing functional foods while ensuring the viability and functionality of probiotic microorganisms during processing and storage is challenging. The survivability of a probiotic is not solely dependent on temperature; it involves a complex interaction between the food matrix, processing conditions, and the specific microbial strain. It is important to note that the stability, safety, and effectiveness of a probiotic are strain-specific, rather than being determined by the species or genus.

Several key factors influence the development of functional foods containing probiotics. Seven primary characteristics are critical when formulating these products: (1) the type of probiotic; (2) the amount of probiotic added; (3) the water activity of the product; (4) the probiotic’s viability during processing; (5) the determination of the cell populations added; (6) storage stability; and (7) sensory acceptability [59]. Additionally, factors such as the food matrix and oxygen content can significantly impact probiotic viability.

Many emerging food products proposed as carriers for probiotics lack comprehensive data on the stability and longevity of the probiotic cultures within the food matrix, as well as how storage conditions and processing affect these probiotics. Table 3 summarizes reviewed studies on *Bacillus* stability in food products, key topics, and major findings. A notable gap in the literature is the uncertainty about whether *Bacillus* spores germinate into vegetative cells or remain in spore form under various conditions. In the subsequent sections of this review, we will address the stability of *Bacillus*, keeping in mind the current lack of clarity on whether these spores germinate within the proposed conditions and, if so, the timing of such germination.

### 4.1. Effect of Food Matrix

The food matrix plays a crucial role in the survival of probiotic vegetative cells during food processing [8]. Components of the food matrix, such as proteins, carbohydrates, salt, and flavoring agents, can significantly influence probiotic efficacy and viability. For instance, salt (NaCl) can inhibit probiotic viability; *B. subtilis* remains viable at NaCl concentrations of up to 7–10%, while *B. coagulans* is not viable at concentrations of 5% NaCl or higher [14]. Selecting appropriate food matrix components is essential, as they impact the viability of probiotics within the product [63].

Incorporating probiotics into novel food matrices while minimizing viability loss during shelf life presents a significant challenge [64]. Each component of the food matrix can affect the viability of specific probiotic strains, thereby determining the overall quality of the probiotic product. Although the effects of the food matrix on vegetative probiotics are well documented, its impact on spore-forming probiotics is less clear. It is hypothesized that the enhanced stability of spore-forming probiotics could facilitate their integration into a broader range of food matrices, offering food manufacturers greater flexibility in product formulation.

#### 4.1.1. Carrier Matrices

The effects of carrier matrices on *Bacillus* spores remain largely unexplored. An effective food matrix must protect, carry, and deliver probiotics through various processing and storage conditions to ensure their viability until they reach the gastrointestinal (GI) tract. Common matrices for lactic acid bacteria (LAB), such as *Lactobacillus*, include fermented products. These matrices are advantageous because they buffer stomach acidity and enhance probiotic viability [5]. Dairy products are another common matrix for vegetative cells, largely due to lactose, a major disaccharide that serves as a substrate for probiotics, potentially boosting their viability [5]. Cheese and milk products are particularly effective carriers as they have higher pH levels compared to other dairy products. Additionally, the gel structure of cheese reduces exposure to detrimental factors [27]. For instance, cheddar cheese containing LAB probiotics has been shown to remain stable at 1 × 10^8^ CFU/g after 8 months of ripening [5], and probiotic viability in milk is stable for up to 4–6 weeks under refrigerated conditions [5].

Other matrices can also support probiotic delivery. Ice cream, meat products, and juices are additional carriers. Meat, such as meat sausages, can encapsulate probiotics, protecting vegetative cells [5]. However, ice cream and juices present more challenges for maintaining probiotic viability. Ice cream can form ice crystals that rupture bacterial cell membranes, while juices often have high acidity, which can decrease probiotic viability. Spore-forming probiotic strains, such as *Bacillus coagulans* (GBI-30, 6086) (BC30™), are better suited to these stressors. BC30™ spores are more resistant to acidity compared to vegetative cells and have shown higher viability in products like juices. Studies indicate that BC30™ maintains its viability in yogurt and juice even after digestion [10]. This suggests that both juices and yogurt are suitable carriers for this probiotic strain. Spore-forming bacteria, due to their increased stability, may offer food manufacturers greater flexibility in incorporating probiotics into a wider range of food matrices.

#### 4.1.2. Carbohydrates

Individual components within a food matrix can significantly influence the viability of probiotics particularly when delivered as vegetative cells. There are conflicting perspectives regarding the impact of carbohydrates and probiotic viability.

On one hand, carbohydrates are often considered beneficial as they can act as protection agents that support probiotic growth [5]. Carbohydrates that are easily metabolized are known to enhance probiotic viability under acidic conditions, and they can improve viability during freezing and thawing [5].

Conversely, certain carbohydrates can negatively affect probiotic viability. For example, fructose, sucrose, and glucose have been shown to reduce the viability of *Lactococcus lactis* probiotics, whereas lactose, xylose, and galactose did not have a significant impact [65]. Additionally, high concentrations of sugar in milk have been found to inhibit *Lactobacillus acidophilus* MJLA1 and *Bifidobacterium* spp. BDBB2 [66].

Different strains of lactic acid bacteria exhibit varying carbohydrate preferences, which should be considered when developing functional food products [65]. While these findings are relevant for vegetative bacterial probiotics, spore-forming probiotics such as *Bacillus* may be less affected by these conditions. However, there is currently a lack of research investigating how carbohydrate types influence the viability of spore-forming probiotics.

#### 4.1.3. Fat Content

It has been hypothesized that higher-fat foods, such as chocolate and peanut butter, may enhance probiotic stability despite their slightly elevated water activity [3]. Specifically, peanut butter has been shown to protect probiotics from the adverse effects of other food ingredients. For instance, a study comparing peanut butter-containing products with controls found that more vegetative probiotic cultures survived in the peanut butter formulation than in the control group [67]. Additionally, research on various commercial probiotic mixtures, including *Lactobacillus* and *Bifidobacterium* strains, demonstrated that these strains retained their viability for up to 12 months when stored in peanut butter at 4 °C [67].

Higher fat content is generally associated with better probiotic protection and stability. For example, a study revealed that increased fat and whey protein levels significantly improved the viability of three *Bifidobacterium* strains [68]. Conversely, another study comparing full-fat and reduced-fat peanut butter found no significant difference in probiotic survival between the two types, challenging earlier findings [69].

These conflicting results suggest that while higher fat content might benefit probiotic stability in some contexts, its impact on *Bacillus* spore probiotics versus vegetative cells remains unclear. Further research is needed to determine whether fat content significantly influences the survival of *Bacillus* spores or if it primarily affects vegetative probiotics.

### 4.2. Effect of Water Activity

Several reports highlight water activity (aw) as a crucial factor influencing the stability of vegetative probiotics [3,5]. Water activity measures the amount of free water available for microbial growth in a product. A study evaluating the long-term stability of *Lactobacillus* in dry food matrices such as flaxseed, maltodextrin, and laboratory sand assessed three different water activity levels: 0.11, 0.22, and 0.43. The findings revealed that higher water activity correlated with reduced probiotic viability. Specifically, at 0.43 aw, there was a 2.4 log reduction in probiotic viability, whereas at 0.11 aw, the reduction was only 0.29 logs. This pattern was consistent across the different food matrices, suggesting that probiotic stability is more influenced by water activity than by the specific food matrix [64].

In a separate study involving dried banana slices, *Lactobacillus rhamnosus* exhibited a 1.5-fold higher inactivation rate at intermediate water activity (0.55–0.77) compared to low water activity (0.11–0.33) [70]. The probiotic remained viable for up to 28 days at lower water activity but only about 15 days at higher levels [70]. Generally, for traditional probiotic-containing foods, a low water activity combined with moisture-resistant packaging is essential for extended shelf life, with an aw below 0.25 required to ensure 12 months of stability at 25 °C [3].

While these studies address the effects of low and intermediate water activity, the impact of higher water activity levels is less explored. Furthermore, research on water activity has predominantly focused on vegetative cells rather than *Bacillus* spores. Spores may offer greater stability than vegetative cells, potentially improving survival in products with higher water activity. As maintaining probiotic viability in high-water-activity products remains challenging for traditional probiotics, *Bacillus* spore preparations could provide enhanced stability in such conditions.

### 4.3. Effect of Food Processes

Probiotics can be highly sensitive to various food processing conditions, which may include high temperatures, the nature of the food matrix, or a combination of both. Common food processing techniques such as baking, drying, boiling, microwaving, and deep-fat frying can adversely affect the viability of probiotics when they are incorporated into food products. To ensure the efficacy and benefits of probiotic products, it is essential to assess the stability and survival of spore-forming probiotics under these processing conditions before incorporating them into food products.

#### 4.3.1. Drying

Drying involves removing water from food products through evaporation to achieve specific textures or to inhibit the growth of mold and bacteria. The food industry employs various drying methods to address different needs [71]. In the context of probiotic foods, such as dried fruit snacks, drying presents challenges related to maintaining probiotic viability. Factors like water removal, high temperatures, and exposure to oxygen during the drying process can negatively impact probiotic survival [20].

A study on dried apple cubes enriched with *Lactobacillus plantarum* revealed that the drying process significantly affects probiotic survival, with freeze-drying emerging as the most effective method for preserving probiotic viability. This is because freeze-drying operates at much lower temperatures compared to other drying methods, thereby better preserving temperature-sensitive probiotics [71].

Osmotic dehydration has been employed in many dried fruit products to mitigate high processing temperatures. Although the success has been limited, research involving freeze-dried bananas inoculated with probiotics and assisted by osmotic dehydration has shown promise. Osmotic dehydration reduces water content before the drying process, which helps maintain probiotic viability while also enhancing the product’s sensory, nutritional, and functional properties [70]. In this study, *Lactobacillus rhamnosus* in freeze-dried samples remained stable for up to 20–28 days across various water activity levels (0.115 to 0.327 a_w_) [70].

These findings suggest that probiotic products can be successfully created through drying using *Lactobacillus*. However, there is currently no research on how different drying methods might affect *Bacillus* strains, indicating a need for further investigation.

#### 4.3.2. Baking

Baked products have been highly challenging for the delivery of probiotics because of the high temperatures required during the baking process, which often lead to substantial microbial inactivation [72]. A study exploring the possibility of adding probiotics to bread was conducted, in which *Bifidobacterium lactis* (Bb12) was added to the bread dough before the baking process [25]. Breads were baked at 165 °C, 185 °C, and 200 °C for 0, 3, 6, 9, and 12 min. The viable count of probiotics decreased slightly after 3 min of baking and then reduced substantially from 3 to 6 min of baking at all the temperatures explored [25]. Baking temperature had a significant difference in the viability of the probiotic. The loaves of bread baked at 205 °C had the largest reductions in viability while those baked at 165 °C had the lowest reductions. Overall, this probiotic was not strong enough to survive in baking temperatures and prevent thermal death during baking.

A further study explored the viability of *Lactobacillus plantarum* during the bread-baking process [23]. The bread was baked at 175 °C, 200 °C, and 235 °C for 8 min, and the viability of bacteria was determined every 2 min at the crust and crumb of the bread [23]. Under all baking conditions, the viability of probiotics decreased from 10^9^ logs to between 10^5^ and 10^4^ logs after baking [23]. It was determined, yet again, that this specific probiotic strain is not suitable for probiotic bread without protective measures such as encapsulation.

High temperatures are needed in baking and other food processes. As a result, baking temperatures lead to the inactivation of probiotics like *Lactobacillus* and *Bifidobacterium*. Alternatives such as microencapsulation and edible films have been used to combat this issue, but more cost-effective alternatives should be found. This makes baked goods an ideal product category for the use of *Bacillus* probiotics because they can survive more severe baking temperatures.

#### 4.3.3. Other Food Processes

Baking and drying are not the only food processes that impact probiotic viability. Other methods, such as boiling, microwaving, frying, pasteurization, and more, can also significantly affect the stability of probiotic bacteria. A study by [61] investigated the effects of various food processing conditions on probiotic viability using eight different *Bacillus* strain spores. The processing conditions included pasteurization, cooking, baking, drying, fermentation, irradiation, extrusion, and supercritical carbon dioxide [61].

The study found that probiotic viability varied significantly across these conditions. Most treatments resulted in reductions exceeding 2 logs in probiotic spore viability, with irradiation causing up to 4.9 logs of reduction [61]. However, four of the tested *Bacillus* strains, including *Bacillus subtilis* Bn1 and *Bacillus coagulans* GBI-30, demonstrated greater resistance to these processing conditions. Notably, *B. coagulans* GBI-30 exhibited superior resistance to most of the tested conditions.

These results underscore the importance of strain specificity when selecting *Bacillus* spores for probiotic applications. The choice of spore strain should consider not only the food matrix but also the specific processing conditions it will endure.

Researchers investigated the impact of household cooking methods on the viability of *Bacillus* spores in cooked sausage [15]. The study examined three cooking methods—boiling, microwaving, and deep-fat frying—and two probiotic strains: *Bacillus subtilis* var. *Natto* ATCC 15245 and *Bacillus coagulans* ATCC 31284 [15]. Additionally, sausages were prepared with varying meat contents: 40% red meat, 55% red meat, 70% red meat, and 55% chicken meat.

The results revealed significant differences in spore viability across the cooking methods. Boiling yielded the highest viability, with spore counts ranging from 8.48 to 9.11 logs CFU/10 kg. This was followed by microwaving (7.92 to 8.95 log CFU/10 kg) and deep-fat frying (8.76 to 7.63 log CFU/10 kg) [15]. The meat content in the sausages also notably affected spore viability. Higher meat concentrations led to increased extraction of salt-soluble proteins, which, in combination with added salt and phosphate, contributed to greater stabilization of the spores within the sausage batter [15].

Furthermore, the specific strain of *Bacillus* spores showed varying levels of viability depending on the sausage formulation and cooking method. The interaction between the probiotic strain, the type of sausage formulation, and the cooking method significantly influenced the overall viability of the probiotic.

Other food processes, such as boiling and pasteurization, also involve high temperatures akin to baking. Interestingly, studies have shown that the effect of temperature on the viability of different *Bacillus* strain spores can be non-linear and unpredictable [10]. For instance, one strain exhibited a nearly 3 log reduction in viability during juice pasteurization at 95 °C, yet only a 1 log reduction during baking at 180 °C, potentially due to the acidic environment of the juice. Conversely, another strain that showed less than a 0.4 log reduction during milk pasteurization at 65 °C experienced a greater than 2 log reduction during drying at the same temperature [10].

This variability suggests that survivability is not merely a function of process temperature but rather a complex interplay between the product matrix and processing conditions. For example, a study on the inclusion of *Bacillus coagulans* spores in enriched pasta demonstrated that the spores survived both the pasta-making and cooking processes, with minimal reductions in microbial counts after boiling for 5–7 min [12]. However, longer boiling times (9–11 min) resulted in significantly greater losses, indicating that both temperature and duration are crucial factors for probiotic viability. Thus, a deeper understanding of how *Bacillus* spores survive under varying processing conditions is essential for optimizing their use in food products.

Another study [14] examined the viability of probiotic spores under various processing and storage conditions. In this research, probiotics were inoculated into sausages both before and after processing and then stored at refrigerated temperatures for up to 45 days. The study found no significant decrease in the viability of *Bacillus* spores when the sausages were inoculated after processing. Specifically, *Bacillus subtilis* var. Natto ATCC 15245 decreased from 9.82 to 8.50 logs CFU/g, and *Bacillus coagulans* ATCC 31284 decreased from 9.26 to 8.78 logs CFU/g [14].

In contrast, sausages inoculated before processing showed significant differences between the two probiotics. *Bacillus coagulans* experienced notable reductions in viability after heat treatment, while *Bacillus subtilis* showed significant declines throughout the refrigerated storage period. Despite these reductions, both spore types maintained adequate microbial counts to be classified as probiotic food products, with a 3–4 log reduction observed following the cooking step [14].

This study underscores the strain-specific nature of *Bacillus* spores, a finding consistent with [61]. It further highlights the importance of strain-specific responses to processing and storage conditions, demonstrating that while one strain may lose viability under certain conditions, another may remain stable.

### 4.4. Effect of Storage Conditions

After food processing, the challenge of maintaining probiotic viability during storage becomes critical. The loss of probiotics during storage often surpasses the loss incurred during processing [20]. While traditional probiotic products like yogurt typically have a shelf life measured in weeks, many new functional foods are designed for longer shelf lives, extending to months or even years. Unlike dairy-based probiotics, which are usually refrigerated, many of these new products are intended to be shelf-stable, which introduces new challenges for maintaining probiotic viability.

Storage conditions such as freezing can damage vegetative cells due to ice crystal formation [5]. Refrigerated storage is commonly recommended for probiotic products, but this comes with high transportation and storage costs and the risk of probiotic loss if temperatures are not strictly maintained [73]. Indeed, storage temperature is a major factor affecting the viability of vegetative probiotics. A study by [67] found that as storage temperatures rise, the mortality rate of vegetative probiotic cultures increases. Elevated temperatures can lead to nutrient depletion and reduced viability of probiotics. For prolonged shelf life, cool temperatures and low water activities are essential for maintaining the viability of most vegetative probiotics [63].

Conversely, research by [69] demonstrated that certain probiotics, including strains of *Streptococcus*, *Lactococcus*, *Lactobacillus*, and *Bifidobacterium*, can remain stable at room temperature in food products like probiotic peanut butter. This product, available in both full-fat and reduced-fat options, showed only minor decreases in probiotic viability (less than 0.1 log CFU/g) after 48–52 weeks [69]. This suggests that while extreme temperatures adversely affect vegetative probiotics, some strains can maintain stability under less stringent conditions.

Most research on storage conditions has focused on vegetative bacteria, with less attention given to spore-forming probiotics. Studies by [11,13] on *Bacillus coagulans* MTCC 5856 and *Bacillus coagulans* GBI-30 6086 during frozen storage revealed that *B. coagulans* MTCC 5856 retained its viability for up to 12 months, unlike *B. coagulans* GBI-30 6086 [11,13].

Given the limited research on the impact of storage conditions on *Bacillus* spore viability, further studies are needed to better understand their stability. This knowledge will be valuable for developing new functional probiotic products.

## 5. Sensory Acceptability

Sensory tests are crucial for assessing whether the inclusion of functional ingredients such as probiotics impacts consumer acceptability. Sensory properties significantly influence the overall appeal of probiotic-enriched foods and thus drive consumer acceptance [5]. However, it remains how adding probiotics to various food matrices affects sensory acceptability. Research results are mixed: some studies suggest that probiotics can negatively impact the quality and sensory properties of food products, while others report no adverse effects [16,27]. The production of lactic acid by probiotics which can alter taste and aroma, might contribute to these sensory changes [27]. Despite this, research in this area is still limited.

For example, a study investigating the sensory acceptability of dark chocolate with added *B. coagulans* found no adverse effects on the sensory or nutritional qualities of the product [16]. Similarly, research by [28,74] indicated that cereal bars containing probiotics (such as *Lactobacillus* and *Bifidobacterium*) were received by consumers with the same level of approval as those without probiotics, showing no significant impact on sensory attributes. In contrast, a study by [17] reported that adding *B. coagulans* spores led to significant differences in flavor and overall acceptability, with sensory scores inversely related to the number of spores added.

## 6. *Bacillus* in Food Products

Although many factors affect probiotic efficacy, certain food formats effectively incorporate probiotics and enable them to deliver their proposed health benefits. Numerous products on the market now contain *Bacillus*, as detailed in Table 4. However, it remains uncertain whether these probiotics are consistently retained in these products. *Bacillus* probiotics can be integrated into a wide range of food matrices, including apple sauce, probiotic tea, and chocolate (Table 4). The versatility of these spores in various food products illustrates a significant advancement for food manufacturers, offering new opportunities for probiotic inclusion in diverse dietary options.

## 7. Conclusions

There is an increasing desire to create new products with probiotic properties. *Bacillus* has enormous potential to be incorporated into different food products. Each product has different requirements and so do *Bacillus* probiotics. Food companies can incorporate these by choosing the right strain for their specific product. Specifically related to the stability of *Bacillus* probiotics, there are various avenues to be explored. New *Bacillus* strains are being manufactured and are FDA-approved, but little is known about their survivability and viability in food processing and storage conditions. Conducting this research will determine which probiotics are more viable in which processing conditions.

This review highlights the increasing need for developing probiotic-enhanced products, with *Bacillus* strains standing out as an addition due to their versatility and viability in harsh conditions and varying food types. The stability of *Bacillus* spores remains a critical area for exploration, as emphasized in this review. The knowledge of *Bacillus* spores’ viability and effectiveness in different processing and storage conditions is limited. This awareness is important for the food industry and food manufacturers to understand how factors impact *Bacillus* spores’ viability and food product integrity. Furthermore, understanding the environmental factors that affect *Bacillus* spores, such as pH, temperature, and oxygen levels during processing and storage, will be crucial to the food industry. The future research topics identified in this review will enable food manufacturers to optimize the formulation and processing of probiotic products, ensuring that these products retain their efficacy and health benefits throughout their shelf life. By researching *Bacillus* spores’ probiotic viability and stability, the food industry can meet consumer demands for innovative and effective probiotic functional foods.

## Figures and Tables

**Table 1:** Health benefits and corresponding mechanisms of action by *Bacillus* probiotic bacteria **.

Beneficial Effects to the Host	Mechanisms by Probiotic
1. Competitive inhibition of pathogens within GI tract	Both Indirect and direct inhibition by means of changing pH, competition for oxygen/nutrients, and production of bacteriocins.
2. Improvement of nutritional value	Synthesis of vitamins and co-factors
3. Relief of dietary intolerance	Catabolism of dietary ingredients
4. Anti-inflammatory and immune-modulating effects	Stimulating the production of anti-inflammatory cytokines Increasing both the number and activity of T-cells
5. Improvement of gut function	Relief of symptoms associated with irritable bowel syndrome Support for the integrity and function of the gut barrier
6. Reduction of alleries	Suppression of hypersensitivity of allergens
7. Reduced risk of cardiovascular disease	Lowering cholesterol levels through the deconjugation of bile salts Generation of peptides that reduce hypertension
8. Reduced risk of cancer	Detoxification of carcinogenic metabolites

** Modified from [36].

**Table 2:** Examples of commercial *Bacillus* probiotics and probiotic supplements that are available on the market.

Species Component(s)	Company/Product Name	Company Location	*Bacillus* Strain Name(s)
*B. cereus*	Mega Labs	Rio de Janeiro, Brazil	Biovicerin^®^
*B. clausii*	Sanzyme Biologics	Hyderabad, India	SNZ 1971
	Advanced Enzyme Technologies Ltd.	Thane, India	088AE
	Unique Biotech Limited	Hyderabad, India	UBBC-07^®^
	Enterogermina	Varies based on region	Enterogermina^®^
	Synergia Life Sciences (Novonesis)	Bagsværd, Denmark	SC109
*B. coagulans*	Kerry ProActive Health	Tralee, Ireland	GanedenBC30, 6086
	Sanzyme Biologics	Hyderabad, India	SNZ 1969
	Thorne	New York, NY, USA	MTCC 5856
	Advanced Enzyme Technologies Ltd.	Thane, India	DSM 17654
	Mitsubishi-Kagaku Foods Corporation	Tokyo, Japan	SANK 70258
	Sabinsa Corporation	East Windsor, NJ, USA	SBC37-01
	Unique Biotech Limited	Hyderabad, India	Unique-IS2^®^
	Solaray	Salt Lake City, UT, USA	UNKNOWN
	Nature’s Sunshine/NutriBiome	Lehi, UT, USA	MTCC 5856
	Swanson Health Products	Fargo, ND, USA	ProDura
	Sporulac^®^	Haryana, India	SNZ 1969
	LactoSpore^®^	East Windsor, NJ, USA	MTCC 5856
	Synergia Life Sciences (Novonesis)	Bagsværd, Denmark	SC208
*B. licheniformis*	Synergia Life Sciences (Novonesis)	Bagsværd, Denmark	SL307
*B. subtilis*	Deerland Probiotics	Kennesaw, GA, USA	DE111^®^
	Sanzyme Biologics	Hyderabad, India	SNZ 1972
	SporeGen Ltd.	London, United Kingdom	SG188
	BIO-CAT Microbials, LLC	Shakopee, MN, USA	BS-MB40 PTA-122264
	Advanced Enzyme Technologies Ltd.	Thane, India	ATCC SD-7280
	Danisco USA, Inc.	Thomson, IL, USA	Bss-19
	Bio-Kult^®^	Manchester, United Kingdom	PXN 21
	Gnosis by Lesaffre	Marcq-en-Baroeul, France	LifeinU ™ BSCU1
	Kemin/Clostat	Des Moines, IA, USA	PB6 (ATCC PTA-6737)
	Bioprogress Pharma SPA	Anagni, Italy	Domuvar^®^
	Mekophar	Ho Chi Minh, Vietnam	Subtyl
	Lallemand Health Solutions	Quebec, QC, Canada	Rosell^®^-179
	Novonesis (Formerly Novozymes)	Bagsværd, Denmark	ProSilience™ HU58™
Mixed species: *B. indicus*, *B. coagulans*, *B. clausii*, and *B. subtilis*	Just Thrive	Park Ridge, IL, USA	*Indicus* (HU36), *Coagulans* (SC-208), *Clausii* (SC-109), and *Subtilis* (HU58)
	Microbiome Labs/Synbiotic Soft Chews	Orland Park, IL, USA	
Mixed species: *B. licheniformis*, *B. indicus*, *B. subtilis*, *B. clausii*, and *B. coagulans*	Microbiome Labs/MegaSporeBiotic	Orland Park, IL, USA	*Subtilis* (HU58), *Indicus* (HU36), *Coagulans* (SC-208), *Licheniformis* (SL-307), *Clausii* (SC-109)
Mixed species: *B. subtilis* and *B. licheniformis*	Biofarma	Kyiv, Ukraine	Biosporin^®^
	CHR Hansen	Hørsholm, Denmark	BioPlus 2B
Mixed species: *B. subtilis* and *B. coagulans*	Jarrow Formulas	Los Angeles, CA, USA	*Subtilis* (DE111™) and *Coagulans* (MTCC 5856)
Mixed species: *B. subtilis*, *B. clausii*, and *Saccharomyces Boulardii*	Microbiome Labs/RestoFlora	Orland Park, IL, USA	*Subtilis* (HU58), *Clausii* (SC109)
Mixed species: *B. subtilis*, *B. clausii*, *Saccharomyces Boulardii*, and *B. coagulans*	Silver Fern Brand ™/Ultimate Probiotic	Salt Lake City, UT, USA	*Subtilis* (HU58), *Clausii* (SC109), *Coagulans* (SC-208)
Mixed species: *B. subtilis*, *B. coagulans*, *Lactobacillus* (*acidophilus*, *rhamnosus*, *salivarius*, *casei*, *plantarum*), and *Bifidobacterium* (*lactis*, *longum*, *breve*)	Ascendant Nutrition Probiotic/Flora 30	San Diego, CA, USA	*Subtilis* (DE111™)
Mixed species: *B. subtilis*, *Lactobacillus* (*acidophilus*, *rhamnosus*, *casei*, *plantarum*), and *Bifidobacterium* (*longum*, *breve*)	The Inner Health Probiotic	Miami, FL USA	*Subtilis* (DE111™)

**Table 3:** Journal articles that examine the stability of *Bacillus* strains in various food products.

*Bacillus* Strain(s)	Food Product(s)	Summary and Major Findings	Reference(s)
*Bacillus. coagulans* (ATCC 31284) and *Bacillus subtilis* (ATCC 15245)	Cooked sausages	*B. coagulans* ATCC 31284 and *B. subtilis* ATCC 15245 were added to sausages to assess the impact of cooking on these probiotics. The cooking process resulted in a 3–4 log reduction in the viability of both probiotic strains. Despite this reduction, the sausages maintained a probiotic concentration of >10^6^ CFU/g during subsequent storage. This study demonstrated that these probiotic strains could remain viable during both the cooking process and the storage of sausages.	[14,15]
*Bacillus coagulans* (ATCC 7050)	Processed cheese	Cheese was inoculated with *B. coagulans* ATCC 7050 and subjected to a consumer sensory test alongside control samples. The results indicated that the addition of probiotics influenced both the flavor and overall liking of the cheese. Specifically, higher levels of probiotics were associated with lower sensory scores, suggesting that increased probiotic content adversely affected the sensory attributes of the cheese.	[17]
*Bacillus coagulans* (MTCC 5856)	Tea and coffee	*B. coagulans* MTCC 5856 was evaluated for spore stability during the brewing of tea and coffee. In its powdered form, *B. coagulans* maintained 99% viability for up to 24 months when stored at room temperature in tea and coffee powder. The strain demonstrated over 94% survivability in both unroasted green coffee and tea after brewing. Consumer tests indicated that the sensory characteristics of the inoculated products were comparable to those of non-inoculated products.	[19]
*Bacillus coagulans* (MTCC 5856)	Banana muffins, waffles, ground coffee, chocolate fudge frosting, hot fudge topping, peanut butter, strawberry preserve, vegetable oil, and glucose syrup	*B. coagulans* MTCC 5856 was tested across various food matrices and processes to evaluate its stability. The probiotic strain demonstrated high stability, maintaining viability for 6–12 months at room temperature. When stored frozen at −20 °C, *B. coagulans* retained its viability for up to 12 months. Additionally, the strain remained viable during high-temperature processes such as baking and coffee brewing.	[11]
	Pasta	Pasta inoculated with *B. coagulans* (BC30) was subjected to boiling for 5, 7, 9, and 11 min. The probiotic strain survived both the pasta-making and boiling processes. The viability was higher with shorter boiling times (5–7 min), while longer boiling times (9–11 min) led to a significant reduction in probiotic viability. Thus, shorter cooking durations resulted in better retention of probiotic viability.	[12]
*Bacillus coagulans* (GanedenBC30, 6086)	Chrysanthemum cookies, egg pastry cakes, mooncakes, muffins, polo bread, soda cookies, sponge cakes, toast	*B. coagulans* (GanedenBC30 6086) was evaluated for its viability in various baked products during baking and up to 15 days of storage. The viability of the probiotic significantly decreased with prolonged storage time. Products stored at room temperature exhibited a faster decline in viability compared to those kept under refrigeration. After 12 days, only three products maintained over 50% viability in refrigerated conditions, while just two products retained similar viability at room temperature. This study indicated that *B. coagulans* is highly sensitive to storage conditions.	[13]
	White and whole wheat bread	This study investigated the resilience of *B. coagulans* (GanedenBC30 6086) throughout the bread-making process and during storage. The evaluation covered various stages of bread production, including mixing, fermentation, and baking, as well as different parts of the bread—crust, crumb, and whole slice. Samples were assessed at four storage intervals: 0, 2, 5, and 10 days. Baking resulted in the most significant reduction in probiotic viability, with reductions exceeding 1.5 log CFU/g. In contrast, mixing and fermentation did not affect probiotic levels. Storage conditions did not contribute to further reductions in viability. Overall, the study demonstrated that bread can effectively serve as a viable carrier for *B. coagulans*.	[18]
*Bacillus coagulans* (GanedenBC30, 6086)	Yogurt and orange juice	*B. coagulans* (GanedenBC30 6086) was incorporated into yogurt and orange juice, and its viability was assessed throughout the stages of digestion. Both yogurt and orange juice exhibited similar log reductions in viability, with reductions of over 1.17 and 0.89 log CFU/g, respectively. Despite these reductions, the results demonstrated that *B. coagulans* maintained significant viability under GI conditions.	[10]
	Dark chocolate	*B. coagulans* was inoculated into dark chocolate to evaluate consumer acceptance compared to uninoculated dark chocolate. The addition of *B. coagulans* did not adversely affect the acceptability or sensory characteristics of the dark chocolate.	[16]
*Bacillus coagulans* (LBSC)	Hot tea (green and lemon), instant coffee, cold lemon tea, cereal, noodle cakes, dark chocolate bars, protein bars, ice cream, chocolate pies, ready-to-drink beverages (cold coffee, mango juice, orange drinks, sports drinks, and coconut water)	*Bacillus coagulans* (LBSC) was incorporated into various food matrices, including drinks, desserts, and convenience foods. This probiotic strain demonstrated over 98% viability both during food processing and under storage conditions. The study confirmed that *B. coagulans* (LBSC) remains stable across a range of food matrices, temperatures, and storage environments.	[60]
*Bacillus flexus* (Hk1), *Bacillus subtilis* (Bn1), *Bacillus licheniformis* (Me1), *Bacillus mojavensis* (KJS3), *Bacillus subtilis* (PXN21), *Bacillus subtilis* (PB6), *Bacillus coagulans* (MTCC 5856), *Bacillus coagulans* (GBI-30, 6086)	Milk, orange juice, meatballs, bread, crystallized pineapple, yogurt, ground black pepper, and spaghetti	This study tested eight different *Bacillus* strains with purported probiotic properties. Among these, strains Bn1, KJS3, Me1, and GBI-30 6086 exhibited the highest resistance, with reductions of less than 1 log CFU/g in most of the tested processes. Generally, these processes caused less than 2 log CFU/g reductions. However, irradiation consistently resulted in the highest log reductions, reaching up to 5 logs CFU/g. Notably, all strains maintained reductions of less than 2 log CFU/g in at least 6 out of 8 tested operations. These results indicate that many of these probiotic strains have significant potential for incorporation into a variety of food matrices and processing conditions.	[61]
*Bacillus subtilis* (HU58 and PXN21)	Whole meal biscuits	Two strains of *B. subtilis* were tested in whole meal biscuits: one human isolate (HU58) and one used in a commercial product (PXN21). Both strains survived baking at 235 °C for 8 min with only a 1 log reduction in viability. These findings indicated that both *B. subtilis* spores effectively withstand the baking process, suggesting high potential for their incorporation into a range of new functional food products.	[62]

**Table 4:** Examples of *Bacillus* probiotic-enhanced food products on the market.

Name of Product	Product Company	Company Location	*Bacillus* Strain(s)	*Bacillus* Name(s)
Apple Sauce + Probiotics	North Coast Organic	Sebastopol, CA, USA	*B. coagulans*	Unique IS2
Breakfast Burrito	Sweet Earth Natural Foods	Moss Landing, CA, USA	*B. coagulans*	GanedenBC30, 6086
Cappuccino	Cooper Moon	Lafayette, IN, USA	*B. coagulans*	GanedenBC30, 6086
Cauliflower Puffs	Vegan Rob’s	Sea Cliff, NY, USA	*B. coagulans*	GanedenBC30, 6086
Cold Brew Coffee	Jus by Julie	Brooklyn, NY, USA	*B. coagulans*	GanedenBC30, 6086
Culture Pop Soda	Culture Pop Soda	Cambridge, MA, USA	*B. subtilis*	DE111
Daily Probiotic Treats	Pet Naturals	Williston, VT, USA	*B. coagulans*	GanedenBC30, 6086
Dandelion Chai Probiotic Tea	Traditional Medicinals	Sebastopol, CA, USA	*B. coagulans*	MTCC 5856
Dark Chocolate Bar	Betty Lou’s	McMinnville, OR, USA	*B. coagulans*	GanedenBC30, 6086
Dark Chocolate Bites	Digestive Advantage	Salt Lake City, UT, USA	*B. coagulans*	GanedenBC30, 6086
Digestion Tea	Vahdam Teas	New Delhi, India	*B. coagulans*	GanedenBC30, 6086
Dried Apricots	Kroger	Cincinnati, OH, USA	*B. coagulans*	GanedenBC30, 6086
Flaxseed Cookies	LesserEvil	Danbury, CT, USA	*B. coagulans*	GanedenBC30, 6086
Frozen Yogurt	Red Mango	Dallas, TX, USA	*B. coagulans*	GanedenBC30, 6086
Fruit and Vegetable Juices	Harvest Soul	Mariette, GA, USA	*B. coagulans*	GanedenBC30, 6086
Granola Bar	Korea’s Granola Bar	Seoul, Korea	*B. coagulans*	MTCC 5856
Happy Belly with Probiotics Drink	Urban Remedy	San Francisco, CA, USA	*B. coagulans*	GanedenBC30, 6086
Health Ade Kombucha	Health Ade	Los Angeles, CA, USA	*B. coagulans*	MTCC 5856
Hemp Protein+ with Flaxseed	Linwoods Health Foods	Armagh, United Kingdom	*B. coagulans*	GanedenBC30, 6086
Herbal Tea	Besunyen Holdings Company Ltd.	Hong Kong, China	*B. coagulans*	GanedenBC30, 6086
Hot Oatmeal Cups	thinkThin^®^	Los Angeles, CA, USA	*B. coagulans*	GanedenBC30, 6086
Instant Coffee	Tipton Mills	Columbus, IN, USA	*B. coagulans*	GanedenBC30, 6086
Juice	Garden of Flavor	Cleveland, OH, USA	*B. coagulans*	GanedenBC30, 6086
KetoPROTEIN	Choice	Hirokawa, Japan	*B. coagulans*	GanedenBC30, 6086
Kombucha	Better Booch	Huntington Park, CA, USA	*B. subtilis*	DE111
Kombucha Iced Tea	FeelGood SuperFoods	Castle Rock, CO, USA	*B. coagulans*	MTCC 5856
Lemon Ginger Tea	Bigelow	Fairfield, CT, USA	*B. coagulans*	GanedenBC30, 6086
Mighty Muffin	Flapjacked	Rancho Cucamonga, CA, USA	*B. coagulans*	GanedenBC30, 6086
Muscle Mac PRO	Muscle Mac^®^	Charleroi, PA, USA	*B. coagulans*	GanedenBC30, 6086
Organic Butter Spread	Melt Organic	Boise, ID, USA	*B. coagulans*	GanedenBC30, 6086
Pet Treats	H3 Essentials	Chicago, IL, USA	*B. coagulans*	GanedenBC30, 6086
Pressed Probiotic Water	Suja	San Diego, CA, USA	*B. coagulans*	GanedenBC30, 6086
ProbiOaties Cookies	BiteMarket	Orange, CA, USA	*B. coagulans*	MTCC 5856
Probiotic Fruit Bars	That’s it	Los Angeles, CA, USA	*B. coagulans*	GanedenBC30, 6086
Probiotic Granola	Purely Elizabeth	Boulder, CO, USA	*B. coagulans*	GanedenBC30, 6086
Probiotic Prunes	Mariani Packing Company	Vacaville, CA, USA	*B. coagulans*	GanedenBC30, 6086
Probiotic Smoothies	Nomva	Santa Monica, CA, USA	*B. coagulans*	GanedenBC30, 6086
Protein and Probiotics Powder	thinkThin^®^	Los Angeles, CA, USA	*B. coagulans*	GanedenBC30, 6086
Protein Balls	SimplyFUEL	Venice, CA, USA	*B. coagulans*	GanedenBC30, 6086
Soft-Baked Snack Bars	BelliWelli	Los Angeles, CA, USA	*B. coagulans*	GanedenBC30, 6086
Sparkling Probiotic Drink	KeVita	Oxnard, CA, USA	*B. coagulans*	GanedenBC30, 6086
Synbiotic Soft Chews	Microbiome Labs	Orland Park, IL, USA	*B. indicus*, *B. coagulans*, *B. clausii*, and *B. subtilis*	*B. indicus* (HU36), *B. coagulans* (SC208), *B. clausii* (SC 109), and *B. subtilis* (HU58)
Tutti Frutti Frozen Yogurt	Tutti Frutti Frozen Yogurt	Fullerton, CA, USA	*B. coagulans*	MTCC 5856
Ultimate Defense Orange Juice	Uncle Matt’s	Clermont, FL, USA	*B coagulans*	Snz 1969
Wellness Shots	Tulua	Huntington Beach, CA, USA	*B. coagulans*	GanedenBC30, 6086
Yovation Ice Cream	Pierre’s Ice Cream Company	Cleveland, OH, USA	*B. coagulans*	GanedenBC30, 6086
YumButter (Nut Butter)	YumButter	Madison, WI, USA	*B. coagulans*	GanedenBC30, 6086
Yumi Probiotique Trail Mixes	Yumi Organics	Westmount, QC, Canada	*B. subtilis*	Rosell-179

## Data Availability

No new data were created or analyzed in this study. Data sharing is not applicable to this article.
